# Appendiceal involvement in pediatric inflammatory multisystem syndrome temporally associated with severe acute respiratory syndrome coronavirus 2 (SARS-CoV-2): a diagnostic challenge in the coronavirus disease (COVID) era

**DOI:** 10.1007/s00247-022-05346-2

**Published:** 2022-04-08

**Authors:** Tejas H. Kapadia, Mohammed T. Abdulla, Rob A. Hawkes, Vivian Tang, Jenny A. Maniyar, Rachel E. Dixon, Amit F. Maniyar, Kirsten M. S. Kind, Emily Willis, Phil Riley, Yousef M. Alwan, Stavros Michael Stivaros

**Affiliations:** 1 Academic Unit of Paediatric Radiology, Paediatric X-ray Department, Royal Manchester Children’s Hospital, Central Manchester University Hospitals NHS Foundation Trust, Oxford Road, Manchester, M13 9WL UK; 2 Paediatric Rheumatology Department, Royal Manchester Children’s Hospital, Manchester, Manchester, UK; 3 Division of Informatics, Imaging, and Data Sciences, School of Health Sciences, University of Manchester, Manchester, UK

**Keywords:** Abdomen, Appendix, Children, Coronavirus disease 2019, Pediatric inflammatory multisystem disease, Severe acute respiratory syndrome coronavirus 2

## Abstract

**Background:**

Many studies on pediatric inflammatory multisystem syndrome temporally associated with severe acute respiratory syndrome coronavirus 2 (PIMS-TS) have described abdominal findings as part of multisystem involvement, with limited descriptions of abdominal imaging findings specific to PIMS-TS.

**Objective:**

To perform a detailed evaluation of abdominal imaging findings in children with PIMS-TS.

**Materials and methods:**

We performed a single-center retrospective study of children admitted to our institution between April 2020 and January 2021 who fulfilled Royal College of Paediatrics and Child Health criteria for PIMS-TS and who had cross-sectional abdominal imaging. We studied clinical data, abdominal imaging, laboratory markers, echocardiography findings, treatment and outcomes for these children. We also reviewed the literature on similar studies.

**Results:**

During the study period, 60 PIMS-TS cases were admitted, of whom 23 required abdominal imaging. Most (74%) were from a Black, Asian or minority ethnic background and they had an average age of 7 years (range 2–14 years). All children had fever and gastrointestinal symptoms on presentation with elevated C-reactive protein, D-dimer and fibrinogen. Most had lymphopenia, raised ferritin and hypoalbuminemia, with positive severe acute respiratory syndrome coronavirus 2 immunoglobulin G antibodies in 65%. Free fluid (78%), right iliac fossa mesenteric inflammation (52%), and significantly enlarged mesenteric lymph nodes (52%) were the most common imaging findings. Appendiceal inflammation (30%) and abnormal distal ileum and cecum/ascending colon wall thickening (35%) were also common. All children responded well to medical management alone, with no mortality.

**Conclusion:**

In addition to free fluid, prominent lymphadenopathy, and inflammatory changes in the right iliac fossa, we found abnormal long-segment ileal thickening and appendicitis to be frequent findings. Recognition of appendiceal involvement as a component of the PIMS-TS spectrum should help clinicians avoid unnecessary surgical intervention as part of a multidisciplinary team approach.

**Supplementary Information:**

The online version contains supplementary material available at 10.1007/s00247-022-05346-2.

## Introduction

Coronavirus disease 2019 (COVID-19), the disease caused by severe acute respiratory syndrome coronavirus 2 (SARS-CoV-2), was initially thought to be a primarily adult disease with a high mortality in older adults and those with associated comorbidities. As the pandemic progressed, however, children exposed to SARS-CoV-2 began to present with an inflammatory syndrome similar to Kawasaki disease, but with a wider spectrum of clinical symptoms and some distinct features such as those seen in older age groups including evidence of cardiac injury [1]. Following national and international expert consensus, the Royal College of Paediatrics and Child Health (RCPCH) in the United Kingdom described a novel syndrome: pediatric inflammatory multisystem syndrome temporally associated with SARS-CoV-2 (PIMS-TS) [2]. This was termed multisystem inflammatory syndrome in children (MIS-C) by the Centers for Disease Control and prevention (CDC) in the United States, with subsequent diagnostic criteria approved by the World Health Organization (WHO) [3] (Online Supplementary Material 1).

Pediatric inflammatory multisystem syndrome was first reported in April 2020 in the United Kingdom (UK) as a hyperinflammatory syndrome in children with a possible association with SARS-CoV-2 [4]. The inflammatory reaction occurs in the latent phase of COVID-19 infection, primarily with cardiovascular and gastrointestinal involvement. According to recent data from the WHO, of the total reported cases around the world across all age groups, children and adolescents <18 years of age account for 8.5% of cases [5]. According to the UK Office for National Statistics, 1.7% of all children (≤16 years) tested positive for COVID-19 between 26 April 2020 and 30 January 2021 [6]. One of the largest early studies published in children with COVID-19 in the UK identified that 11% of the total pediatric COVID-19 cohort progressed to develop multisystem inflammatory syndrome according to the WHO definition [7]. Mortality is generally very low in PIMS-TS, even in children admitted to pediatric intensive care, where the mortality is around 3% [8].

Other than fever, gastrointestinal symptoms are the most common presenting feature of PIMS-TS [7]. Many children present to the emergency department with an acute abdomen suspicious for appendicitis or inflammatory bowel disease. Early in the pandemic, some children underwent surgery for acute abdominal presentations that clinically mimicked acute appendicitis. At surgery, these children were found to have a non-inflamed appendix with peritonitis [9].

This retrospective study from the UK’s largest children’s hospital aims to provide insight into the abdominal imaging features of PIMS-TS and their correlation with clinical presentation, blood laboratory results and treatment outcomes. We elaborate on the involvement of the appendix, mesenteric lymphadenopathy, and small/large bowel inflammatory changes in the abdomen to expand the phenotype of PIMS-TS and to differentiate this entity from non-COVID-related appendicitis and inflammatory bowel disease.

## Materials and methods

Institutional review board (IRB) approval was waived through the current National Health Service COVID exemption. The data were clinically obtained and anonymized; hence, consent to participate was also waived.

### Case collection and selection criteria

We performed an observational retrospective study of all children with PIMS-TS who presented to the Royal Manchester Children’s Hospital from 1 April 2020 to 26 January 2021. We searched the radiology reporting software (Central Data Networks [CDN] Radiology Information System, or CRIS) using the keywords “abdominal pain/vomiting/diarrhoea,” “Kawasaki disease,” “Kawasaki like disease,” “toxic shock syndrome” and “PIMS-TS.” The severity of abdominal pain was classed into three categories (mild/moderate/severe), based on retrospective review of clinical history and examination findings documented in medical notes. Inclusion criteria for PIMS-TS were set according to the published case definition outlined by RCPCH [2] and only those children with some form of abdominal imaging — US, CT or MRI — were included (Online Supplementary Material 2). CT scans performed in these children were part of routine clinical care and no additional CT scan was performed, or additional radiation dose involved in this study.

### Case review

We reviewed the electronic patient record to determine patient demographics (age/gender/ethnicity/body mass index [BMI]) and clinical presentation (Table 1). Laboratory results from the first 3 days of admission were noted with a wider range of findings in children with multiple imaging exams (Online Supplementary Material 3). The following blood tests were documented for each child: C-reactive protein (CRP), ferritin, fibrinogen, D-dimer, lymphocyte count, neutrophil count, albumin, brain natriuretic peptide (NT-proBNP) and troponin-T (Online Supplementary Material 4). Median and interquartile range (IQR) values were calculated for each test, as well as percentage abnormality (Online Supplementary Material 5). SARS-CoV-2 reverse transcriptase-polymerase chain reaction (RT-PCR) (throat/nasal swab) and serology (SARS-CoV-2 antibody) status was documented. We captured data regarding pediatric critical care admission, inotropic support, ventilatory support and any surgical intervention.

**Table 1:** Summary of clinical symptoms in the 23 children included in the study (age range 2–14 years, mean age 7 years)

Symptoms	Number of patients	Percentage affected
Fever	23/23	100%
Abdominal pain	22/23	96%
Vomiting	10/23	43%
Rash	9/23	39%
Conjunctivitis	9/23	39%
Red lips	4/23	17%
Diarrhea	3/23	13%
Central nervous system symptoms^a^	3/23	13%
Cervical adenopathy	3/23	13%
Myalgia	2/23	9%
Respiratory symptoms^b^	2/23	9%

^a^Headache (*n*=2), confusion (*n*=1)

^b^Cough (*n*=1), respiratory distress (*n*=1)

Two consultant paediatric radiologists (T.H.K., V.T.) with a combined experience of 18 years reviewed study images on a diagnostic workstation from the source Digital Imaging and Communications in Medicine (DICOM) imaging datasets. The two derived a consensus opinion on imaging findings. The abdominal imaging findings documented were small bowel/large bowel involvement (specifically ileum/cecum/ascending colon wall thickening; >3 mm thickening was considered abnormal), enlarged and inflamed appendix (a diameter ≥6 mm was considered abnormal) with thick enhancing walls, echogenic mesentery on US or mesenteric fat-stranding on CT/MRI, enlarged mesenteric lymph nodes (>8 mm in short-axis diameter), free fluid in abdomen or a localized collection (Table 2). For children with multi-modality imaging (e.g., both US and CT/MRI), we considered the findings from CT or MRI over US. Additional imaging abnormalities on chest radiograph, CT chest and MRI brain, if performed, were noted. The findings from two-dimensional (2-D) echocardiography reports were noted for systolic/diastolic dysfunction and coronary artery dilatation/aneurysm.

**Table 2:** Abdominal imaging findings observed in 23 children with pediatric inflammatory multisystem syndrome temporally associated with severe acute respiratory syndrome coronavirus 2 (PIMS-TS) compared with pooled data of relevant reviewed studies (*n*=10) following a literature search

Imaging findings on abdominal US/CT/MRI	Incidence in children included in our study (*n*=23)	Average of incidence from pooled data of 10 similar studies in literature^d^
Free fluid	78%	49.6%
Mesenteric inflammation	52%	10.3%
Enlarged mesenteric lymph nodes	52% (RIF)	24.8%
Ileal thickening	35%	18.9%
Cecal/ascending colon thickening	35%	7.8%
Appendicitis	30%	1.9%
Other bowel thickening^a^	18%	2.3%
Renal involvement^b^	22%	8.4%
Splenomegaly	17%	3.1%
Pericholecystic fluid / GB wall edema	4%	13.5%
Collection^c^	4%	–
Esophageal thickening	4%	–
Hepatomegaly	–	10.6%
Splenic lesions/infarction	–	0.9%

*CT* computed tomography, *GB* gallbladder, *MRI* magnetic resonance imaging, *RIF* right iliac fossa, *US* ultrasound

^a^Jejunal thickening (*n*=3), sigmoid thickening (*n*=1)

^b^Echogenic kidneys (*n*=2), enlarged kidneys (*n*=3)

^c^One child had ileal thickening, mesenteric inflammation and enlarged mesenteric lymph node on initial US scan; subsequent follow-up US, CT and MRI showed a collection in the right iliac fossa and possible perforated appendicitis with a collection. This was treated conservatively as a PIMS-TS complication and the inflammation and collection had resolved at further follow-up US

^d^Details of studies from literature included in analysis summarized in Online Supplementary Material 6

### Statistical analysis

Data analyses were done in Excel 2010 (Microsoft, Redmond, WA). Results are presented as numbers and percentages for categorical data and as medians and IQRs for continuous data.

### Review of literature

We searched the literature on PubMed and Scopus using the following keywords: “COVID-19/SARS-CoV-2” and “PIMS-TS/PIMS/paediatric multisystem inflammatory syndrome/MIS-C/multisystem inflammatory syndrome in children/hyperinflammatory syndrome in children/Kawasaki-like disease/toxic shock syndrome” and “children/paediatric and abdominal findings/abdominal imaging/abdominal US/abdominal CT.” The age limit was set to <21 years and we excluded studies with only adults (≥ 21 years). From the initial detailed search, we found 20 studies (case series, case reports, observational studies and cross-sectional studies) that mentioned abdominal findings on imaging or surgery in pediatric patients with PIMS-TS or MIS-C. We included only 10 studies where the abdominal imaging findings were described in at least 5 children (Online Supplementary Material 7). We analyzed the percentage of children undergoing imaging and the imaging findings, and we studied the outcome of COVID-19 testing (RT-PCR or serology). The data extraction was carried out by two authors (T.H.K., M.T.A.), independently reviewed by a third author (V.T.) and any differences were resolved by consensus of these three authors. The categorical pooled data of the 10 studies included in the review are presented in tabular form (Table 2) [4, 10–18] as percentages of the population undergoing abdominal imaging in all studies (e.g., splenomegaly = 5/50 as 10%). We performed a paired *t*-test to look for differences in population demographics between our study and the studies included in the review analysis.

## Results

The radiology reporting software keyword search identified 143 children presenting with abdominal pain, vomiting or diarrhea who required abdominal imaging between 1 April 2020 and 26 January 2021. In the same period, 60 children (fulfilling the diagnostic criteria of PIMS-TS) were admitted to Royal Manchester Children’s Hospital. Following a separate search of both the reporting software and PIMS-TS registry, a total of 23 children (12 boys, 11 girls) diagnosed with PIMS-TS were identified who had had abdominal imaging (US/CT/MRI). These children had an average age of 7 years (range 2–14 years) and 52.1% (12/23) were male. Seventeen of 23 (74%) children were from a Black, Asian or minority ethnic background (according to UK government classification) [19]. There were 15/23 (65%) children with body mass index (BMI) above the 50th centile for age.

### Clinical features

#### Symptoms

All children (23/23) had fever and gastrointestinal symptoms. Other symptoms on presentation were abdominal pain (22/23, 96%), vomiting (10/23, 43%) and diarrhea (3/23, 13%). The majority had mild (39%) to moderate (56%) severity of abdominal pain. Other less frequent symptoms are summarized in Table 1.

#### Timing

Most children were asymptomatic at the time of likely primary infection, subsequently presenting with abdominal symptoms and classic laboratory features of PIMS-TS. Five children (22%) had COVID-19 symptomatology at the time of initial infection, with an average duration of 28 days between the initial infection and hospital admission/imaging for PIMS-TS.

#### Multi-systemic involvement

Eleven of 23 (48%) children had abnormal 2-D echocardiogram; 6/11 (54%) had systolic dysfunction, 4/11 (36%) had coronary artery dilation (>4 mm) and 1/11 (9%) had coronary artery aneurysm (Online Supplementary Material 3). Respiratory symptoms were present in 2/23 children: one had cough with a normal chest radiograph; the other had respiratory distress and showed ground-glass changes with consolidations on chest CT. Two of 23 children had neurologic symptoms and brain MRI was performed in one child with confusion. This MRI showed restricted diffusion in the splenium of the corpus callosum, which has previously been reported in a study of neurologic manifestations of COVID-19 [20].

### Management and outcome

Eleven of the 23 (48%) children required admission to pediatric critical care for hypotension/shock. Eight of the 11 (35% of the study cohort) children required inotropic support and 1 child required mechanical ventilation. The other two children were observed in the critical care unit, but did not require escalation of care. Twelve of the 23 (52%) required ward level care only.

The children were managed by a multidisciplinary team including rheumatology, cardiology, critical care, general pediatrics and general surgery. Their treatment was in line with the UK national consensus management pathway recommendations [21], which include intravenous (IV) immunoglobulin (Ig) and IV methylprednisolone. Those who gave consent were recruited to the Recovery trial and managed according to the Recovery protocol [22]. No children from our series required surgical intervention despite appendiceal involvement (see Discussion). There were no deaths, and all children were followed up for at least 6 weeks after discharge in a multidisciplinary PIMS-TS clinic including cardiology and rheumatology expertise.

### Laboratory results

All children (23/23) had elevated CRP, D-dimer and fibrinogen in the first 3 days of admission. Ninety-six percent had lymphopenia, raised ferritin and hypoalbuminemia. The majority displayed neutrophilia (83%) and significantly raised proBNP (87%). Sixty-one percent had a raised troponin-T. (Individual patient data and a summary of laboratory findings are presented in Online Supplementary Material 4 and 5).

All children except one (22/23) tested negative for SARS-CoV-2 RT-PCR on swab tests. Serological testing for SARS-CoV-2 IgG antibodies was performed in 20 children and 13/20 (65%) were positive for antibodies. Of the children who did not have antibodies tested or who tested negative (10/23), one gave a clinical history consistent with acute SARS-CoV-2 infection 6 weeks prior and two had confirmed positive recent contacts.

### Imaging findings

Of the 60 children with confirmed PIMS-TS who presented at our hospital, 23 required abdominal imaging (17 US, 9 CT and 1 MR abdomen; Online Supplementary Material 7). US was the initial modality of choice, except for five children with complex clinical presentations who underwent CT scanning. It must be noted, though, that US was performed at their referring hospital in all five of these children: one was suspicious for appendicitis, three were inconclusive on US because of diffuse inflammation in the right iliac fossa with non-visualization of the appendix, and one was under treatment for PIMS-TS with sudden acute deterioration. Four children had US at presentation and later underwent CT scan because of an unclear diagnosis: in one case the appendix was not visualized but right iliac fossa inflammatory changes were seen on US and appendicitis was suspected; appendicitis was diagnosed on imaging but clinically PIMS-TS was diagnosed in two cases; in the final case there was worsening of PIMS-TS diagnosed on US. This was followed by a CT scan to look for a collection, which was found and was suspicious for appendiceal perforation. The patient was later monitored by MRI. In three of these cases, CT scan helped to rule out appendicitis in view of multiple enlarged mesenteric lymph nodes (average short-axis diameter 17 mm) with right iliac fossa inflammatory changes, free fluid and bowel thickening.

The most common findings on imaging (US/CT/MRI) were free fluid (18/23, 78%) and right iliac fossa mesenteric inflammation and enlarged mesenteric lymph nodes in 12/23 (52%). The average lymph node short-axis size was 11.9 mm (median 12.5 mm, IQR 8–15.8 mm) and average long-axis size was 19.1 mm (median 17 mm, IQR 15–24 mm).

Abnormal bowel wall thickening (>3 mm) was present in 8/23 (35%) children, mostly involving the distal ileum or cecum/ascending colon. In a few cases multiple ileal segments were involved. Three children (13%) had left-side small bowel (jejunal/ileal) wall thickening with circumferential esophageal wall thickening in one (4%); another child had sigmoid colon wall thickening (4%) with surrounding inflammatory changes.

Appendiceal dilation and inflammation (diameter ≥6 mm, with thick enhancing walls) was seen in 7/23 (30%) cases, although this was always seen with other bowel involvement. The appendix measured 6–9 mm (individual measurements were 6 mm/6 mm/8 mm/7 mm/7 mm/9 mm/9 mm) in diameter in the seven children with abnormal appendix. One child had a 3-mm appendicolith complicated by possible appendix tip perforation with pelvic collection 5 days after admission, as seen on MRI.

Other less frequent imaging findings (Table 2) were splenomegaly (4/23; 17%), two cases of echogenic/heterogeneous kidneys (9%), and three cases where renal enlargement was seen (13%). Two children had documented change in kidney size over two scans, along with a positive correlation with changes in inflammatory blood markers. Only one child with echogenic kidneys had an acute kidney injury score of 3, the remaining four cases with renal changes had normal serum creatinine and no acute kidney injury. None of the cases with renal involvement had fascial edema and there were no parenchymal changes in the cases of enlarged kidneys.

### Review of literature

The commonly occurring findings from the pooled data of the 10 included studies were free fluid (49.6%), mesenteric lymphadenopathy (24.8%), mesenteric inflammation (10.3%), ileal thickening (18.9%), gallbladder wall thickening or edema (13.5%), and hepatomegaly (10.6%) (Table 2). Nearly 45% of patients from the review data underwent abdominal imaging. More than half had positive serology (58.9%) and a quarter (26.5%) had positive RT-PCR (Online Supplementary Material 8). Differences in the population demographics of the reviewed literature and our study did not show statistical significance. The two groups were compared for average age (*P*=0.21), gender (*P*=0.89) and Black, Asian or minority ethnic background (ethnicity) (*P*=0.64).

## Discussion

Pediatric inflammatory multisystem syndrome cases have occurred in waves following peaks of acute SARS-CoV-2 infection from April 2020 until the time of submission of this study. Its association with this novel syndrome is now well established [23]. Most of the published literature to date has described the range of clinical symptoms and multisystem imaging findings in PIMS-TS with respiratory, cardiac, abdominal and neurologic involvement. We present children with PIMS-TS who specifically required abdominal imaging. All the children in our study had fever on presentation (100%) and most had gastrointestinal symptoms (96% abdominal pain, 43% vomiting). This is congruent with many similar studies [4, 17, 24] except for one of the larger case series in which fewer patients (nearly 50%) had gastrointestinal symptoms [1]. Most studies included patients presenting with abdominal symptoms mimicking an acute abdomen, such as acute appendicitis; therefore, recognition of the imaging features that favor a diagnosis of PIMS-TS is essential because the pathways of management for these children are significantly different [25].

From all the children with PIMS-TS at our institute, 38% (23/60) had abdominal imaging because of the clinical presentation of acute abdomen. This is consistent with one of the earliest published case series from the UK [13] with 54% (19/35) of those patients having abdominal US. On a broader comparison, the number of children requiring abdominal imaging in our study is similar to that found in an analysis from the pooled data of 10 similar studies, where an average of 45% of the patients had some form of abdominal imaging (Online Supplementary Material 6 and 8) [4, 10–18, 26].

**Fig. 1:**
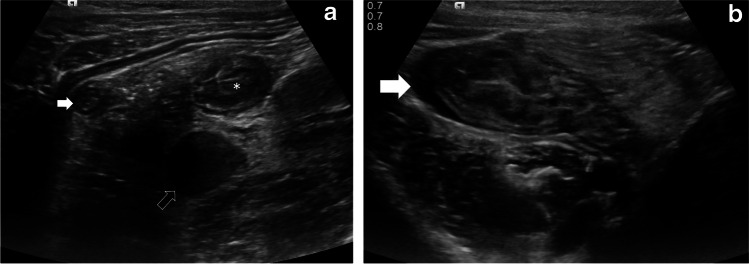
Case 1: US abdomen in a 6-year-old girl who presented with back and right iliac fossa pain. **a, b** Transverse US section of the right iliac fossa. The appendix (*white arrow* in **a**) appears inflamed with a diameter of 6 mm. Note inflamed mesentery, enlarged mesenteric lymph nodes (*black arrow*), thickened distal ileum (*) and cecum (*white arow *in **b**), with free fluid in both paracolic gutters (not shown)

**Fig. 2:**
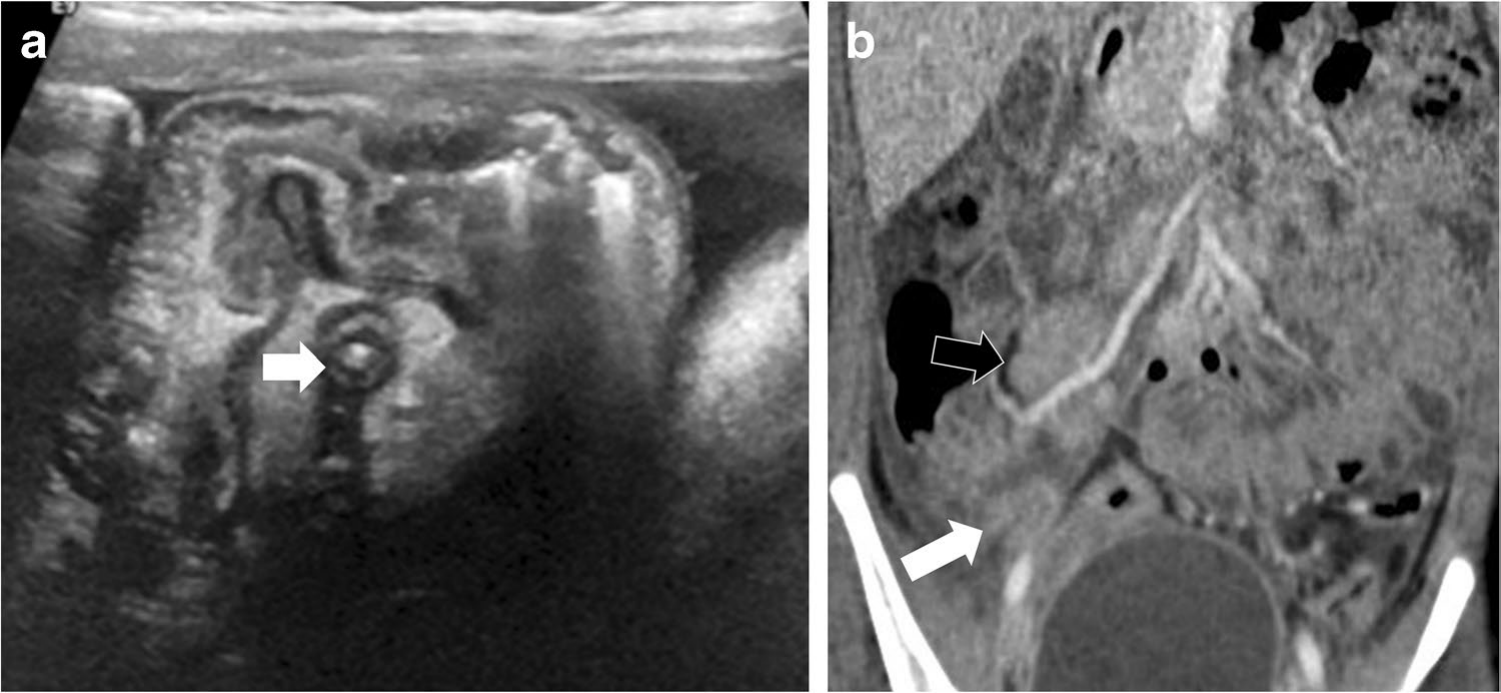
Case 4: An 8-year-old girl presented with abdominal pain, fever, and vomiting. **a** Initial transverse US section of right iliac fossa on presentation shows inflamed appendix (*arrow*) with inflammatory changes. Findings were suspicious for appendicitis. **b** CT scan (coronal post-contrast) shows multiple enlarged ileocolic lymph nodes (*black arrow*), largest 20 mm in short axis, free fluid in right iliac fossa, inflamed mesentery, ileal and cecal thickening and inflamed appendix (8 mm, *white arrow*)

**Fig. 3:**
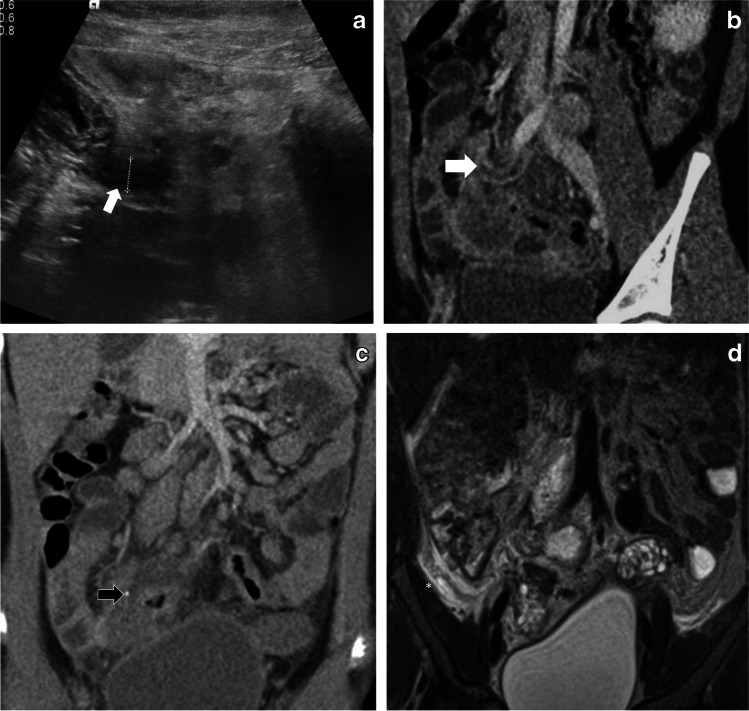
Case 5: A 7-year-old girl presenting with a 3-day history of right iliac fossa pain. **a** Abdominal US transverse section at presentation shows diffuse right iliac fossa inflammation with free fluid and possible appendicitis. **b, c** CT scan (coronal post-contrast) next day confirms extensive right iliac fossa mesenteric inflammation with enlarged lymph nodes, free fluid, thickened appendix (6 mm, *white arrow*), with a small 3-mm appendicolith in the mid segment (*black arrow*) and thickened walls of distal ileum, cecum, proximal ascending colon and sigmoid colon. **d** MRI (T2-W coronal) on day 5 of admission shows similar findings with a small, localized collection in the peri-cecal region (*) with suspicion of a perforated appendix tip. This was followed up on US (not shown), which confirmed resolution of the collection and inflammatory changes after medical management. No surgical intervention was required

**Fig. 4:**
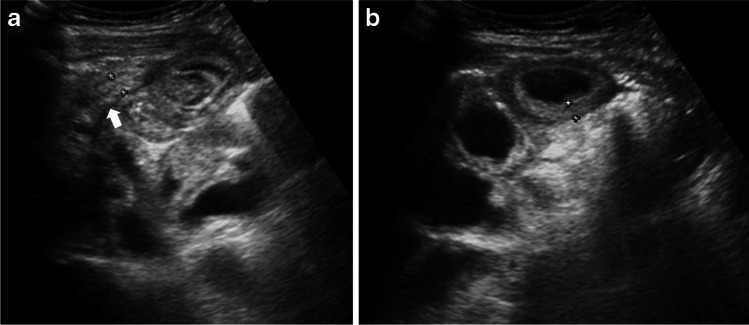
Case 23: a 7-year-old boy with abdominal pain, vomiting and fever. **a, b** US abdomen transverse section of right iliac fossa shows diffusely thickened multiple small bowel loops (*calipers *in **b**) with free fluid and mesenteric inflammation. The appendix is mildly inflamed (7 mm *arrow*). CT scan on day 4 of admission (not shown) showed similar findings as US, in addition to enlarged kidneys which had increased in size compared to initial US

The most common imaging finding in our study was the presence of free fluid, mesenteric inflammation and mesenteric lymphadenopathy (Figs. 1, 2, 3 and 4). We noted that the abnormal nodes were quite prominent, with an average short-axis size of 11.9 mm and average long-axis size of 19.1 mm. Half the children with lymphadenopathy (6/12) had more than six enlarged mesenteric lymph nodes. It is possible that the particularly prominent size of nodes is a phenomenon of PIMS-TS, noting that multiple large lymph nodes are not generally seen in acute appendicitis. Such enlarged lymph nodes can be seen in inflammatory bowel disease (IBD), which generally has small bowel wall asymmetrical thickening (along the mesenteric border), segmental mural enhancement and mesenteric fat expansion [27, 28]; however, the advanced cases of IBD are usually associated with small-bowel stricture, ulcerations and sacculation, fistulas and abscess formation [29].

Abnormal bowel thickening predominantly involving the distal ileum and cecum was observed in a third (35%) of our cases, with an average bowel wall thickness of 5 mm (range 4–7 mm) (Figs. 1 and 4). Some cases also had jejunal (3/23), sigmoid (1/23) and distal esophageal (1/23) involvement. Bowel thickening (especially long-segment ileal involvement) is thought to be much more specific for PIMS-TS and is uncommon in other potential mimics such as acute appendicitis (bowel thickening if present is usually limited to terminal ileum and cecal pole), infective enteritis (asymmetrical thickening of the ileocecal valve, cecum and terminal ileum and enlarged necrotic ileocolic lymph nodes for tuberculous enteritis or symmetrical wall thickening of terminal ileum and most of the colon without involving the surrounding mesenteric fat in acute bacterial enterocolitis) or mesenteric adenitis (surrounding fat-stranding and absence of an underlying cause) [30–32]. A bioinformatics analysis has revealed that angiotensin-converting enzyme 2 (ACE2) receptor expression is significantly higher in the esophagus, ileum and colon [33]. SARS-CoV-2 has a special affinity for ACE2 receptors, and to some extent this explains the propensity for involvement of ileum and colon [34] in our cases.

Comparison of our findings with outcome of the review analysis of pooled data (Table 2) from similar studies shows that the abdominal inflammatory changes were much more common in our study (free fluid, 78% compared to 49%; mesenteric lymphadenopathy, 52% compared to 24%; mesenteric inflammation, 52% compared to 10%; ileal thickening, 35% compared to 18%; and cecum/ascending colon thickening, 35% compared to 8%). An important factor for this difference could be that most of our cases presented during the second wave in the UK when some of the imaging features were already established, compared to many of the studies from the review analysis, which were published from the period of the first wave.

Another important observation in our study is evidence of appendiceal involvement in 7/23 (30%) cases, which is contrary to the current literature on PIMS-TS (Figs. 1, 2, 3 and 4). In a routine clinical setting, acute appendicitis (enlarged appendix ≥6 mm) is commonly associated with peri-appendiceal fat-stranding, focal cecal apical thickening (arrowhead sign), adenopathy, fluid in right paracolic gutter or appendicolith [27]. In our study, all cases with appendiceal thickening also had distal ileum (not limited to ileo-cecal junction) and cecum (circumferential wall thickening extending to involve the ascending colon in some cases) involvement, in addition to mesenteric inflammation, lymphadenopathy, and free fluid (Fig. 4).

The overall assessment of these cases was that although a degree of appendicitis was seen, when interpreted with the other clinical and laboratory findings, a diagnosis of PIMS-TS was favored. All children in our study presenting with acute abdomen had both laboratory tests and imaging at presentation, followed by multidisciplinary team discussion. The multidisciplinary team approach avoided surgery in all our cases, and we strongly recommend such an approach to ensure the best possible management pathway for these children.

We had one complex case (patient 5, Fig. 3) who presented with a 3-day history of high fever, abdominal pain and vomiting. Initial US demonstrated caecal and ileal wall thickening, inflamed appendix (6-mm diameter), multiple enlarged lymph nodes (short-axis 15 mm), free fluid and mesenteric inflammation in the right iliac fossa. PIMS-TS was suggested based on US and clinical presentation, with laboratory tests supporting the diagnosis. CT scan was performed 24 h later, and the findings mirrored the US findings but also showed a small 3-mm appendicolith. MRI performed on day 5 demonstrated a pelvic collection adjacent to the appendix tip with possible appendix perforation. PIMS-TS medical management (IV immunoglobulins and IV methylprednisolone) with continuation of intravenous IV antibiotics led to resolution of inflammatory changes and collection on subsequent US. This was a rare case of appendix perforation secondary to PIMS-TS presenting in the latent phase. The presence of an appendicolith could have been responsible for early perforation in this case. A study of 388 patients with acute appendicitis has shown that appendiceal perforation is more common in the presence of an appendicolith (*P*<0.001) [35].

Early in the pandemic there were a few reports of patients with PIMS-TS having surgical intervention for clinically suspected appendicitis at presentation but found to have a normal appendix intraoperatively [15, 36] who were retrospectively diagnosed with PIMS-TS. In a recent case series, complicated perforated appendicitis was found in 2/4 patients; however, all of them were in the acute phase of the infection (all had negative serology for SARS-CoV-2 and 3/4 had positive RT-PCR), which is contrary to most of the PIMS-TS/MIS-C cases, which present as a delayed immunological reaction, as seen in our study.

While hepatomegaly and splenomegaly have been described, our series included three cases with enlarged kidneys, two of which were shown to fluctuate over time in unison with change in serum inflammatory markers. This is a new finding that might be part of the PIMS-TS spectrum and should be studied in larger multicenter studies.

The 2011 census found that 33.3% of the population from Manchester belongs to a Black, Asian or minority ethnic background group [37]. In our case series, 74% of the children were from Black, Asian or minority ethnic background and 65% had a BMI greater than the 50th centile for age; both these findings correspond to reports in the literature [9, 38, 39].

This study has some limitations. First, the sample size of 23 children is relatively small for wider generalization of our findings; however, the number of children with abdominal imaging (23/60) is significant when compared to similar studies from the literature. In 7 studies included in our review analysis, the number of patients that had abdominal imaging ranged between 6 and 34, with only 2 of these studies including abdominal imaging findings from more than 23 patients (Online Supplementary Material 6). We think there is merit in putting forward our results, particularly because some new findings have been described. We also recognize that because none of the cases with appendiceal involvement had surgical intervention, there are no specimens to prove that the appendix inflammation was not caused by an infective etiology. However, considering the overall imaging, clinical and laboratory findings and the fact that all cases resolved with medical management, we believe that our data strongly support PIMS-TS, rather than acute infection, as the underlying diagnosis in these cases.

## Conclusion

Gastrointestinal symptoms are a common manifestation of PIMS-TS, and abdominal imaging is often vital in facilitating the primary diagnosis of PIMS-TS. However, such imaging must be interpreted in conjunction with clinical and laboratory findings as well as multidisciplinary team discussion. Inflammatory changes in the right iliac fossa, prominent lymphadenopathy, free fluid, abnormal long-segment ileal thickening and involvement of the appendix are not uncommon findings. When present, appendix inflammation usually resolves with PIMS-TS medical management. We believe that appendiceal involvement should be recognized as part of the PIMS-TS spectrum to increase confidence in recognizing PIMS-TS on imaging. A multidisciplinary team approach should be undertaken for all children suspected of having PIMS-TS for best possible outcome.

## Supplementary Information

ESM 1 (DOCX 15.6 kb)

ESM 2 (DOCX 0.99 mb)

ESM 3 (DOCX 22.0 kb)

ESM 4 (DOCX 18.5 kb)

ESM 5 (DOCX 14.1 kb)

ESM 6 (DOCX 17.3 kb)

ESM 7 (DOCX 16.4 kb)

ESM 8 (DOCX 16.6 kb)
