# Supplementary material for: Appendiceal involvement in pediatric inflammatory multisystem syndrome temporally associated with severe acute respiratory syndrome coronavirus 2 (SARS-CoV-2): a diagnostic challenge in the coronavirus disease (COVID) era
Source: Pediatr Radiol. 2022 Apr 8;52(6):1038–47. doi: 10.1007/s00247-022-05346-2 (PMC8990674; doi:10.1007/s00247-022-05346-2)
Supplement: Supplementary file 1 — (DOCX 15.6 kb) [file 247_2022_5346_MOESM1_ESM.docx]

**Online Supplementary Material 1** Case definitions for pediatric inflammatory multisystem syndrome (PIMS-TS) /multisystem inflammatory syndrome in children (MIS-C) from the Royal College of Paediatric and Child Health and the World Health Organization

| Royal College of Paediatric and Child Health (RCPCH) [2] | World Health Organization (WHO) [3] |
| --- | --- |
| 1. A child presenting with persistent fever, inflammation (neutrophilia, elevated C-reactive protein, and lymphopenia) with evidence of single- or multi-organ dysfunction (shock, cardiac, respiratory, renal, gastrointestinal, or neurologic disorder) with additional features. This may include children fulfilling full or partial criteria for Kawasaki disease 2. Exclusion of any other microbial cause, including bacterial sepsis, staphylococcal or streptococcal shock syndromes, infections associated with myocarditis such as enterovirus 3. SARS-CoV-2 PCR testing may be positive or negative | Children and adolescents 0–19 years of age with fever >3 days  **AND** two of the following:   1. Rash or bilateral non-purulent conjunctivitis or muco-cutaneous inflammation signs (oral, hands or feet) 2. Hypotension or shock 3. Features of myocardial dysfunction, pericarditis, valvulitis or coronary abnormalities (including ECHO findings or elevated troponin/NT-proBNP) 4. Evidence of coagulopathy (by PT, PTT, elevated D-dimers) 5. Acute gastrointestinal problems (diarrhea, vomiting or abdominal pain)   **AND**  Elevated markers of inflammation such as erythrocyte sedimentation rate, C-reactive protein, or procalcitonin  **AND**  No other obvious microbial cause of inflammation, including bacterial sepsis, staphylococcal or streptococcal shock syndromes  **AND**  Evidence of COVID-19 (RT-PCR, antigen test or serology positive), or likely contact with patients with COVID-19 |

*COVID-19* coronavirus disease 2019, *ECHO* echocardiography, *NT-proBNP* brain natriuretic peptide, *PT* prothrombin time, *PTT* partial thromboplastin time, *RT-PCR* reverse transcriptase-polymerase chain reaction *SARS-CoV-2* severe acute respiratory syndrome coronavirus 2
