# Supplementary material for: Appendiceal involvement in pediatric inflammatory multisystem syndrome temporally associated with severe acute respiratory syndrome coronavirus 2 (SARS-CoV-2): a diagnostic challenge in the coronavirus disease (COVID) era
Source: Pediatr Radiol. 2022 Apr 8;52(6):1038–47. doi: 10.1007/s00247-022-05346-2 (PMC8990674; doi:10.1007/s00247-022-05346-2)

**Online Supplementary Material 2** Inclusion and exclusion of cases in our study. *PIMS-TS* pediatric inflammatory multisystem syndrome, *RCPCH* Royal College of Paediatrics and Child Health


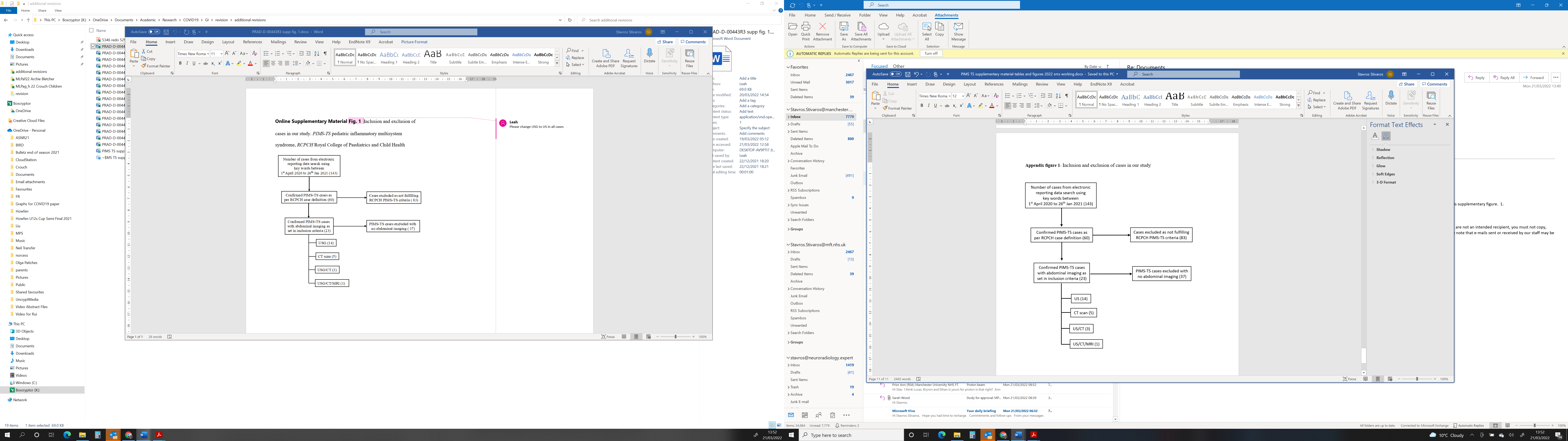

Supplement: Supplementary file 2 — (DOCX 0.99 mb) [file 247_2022_5346_MOESM2_ESM.docx]
