# Supplementary material for: Appendiceal involvement in pediatric inflammatory multisystem syndrome temporally associated with severe acute respiratory syndrome coronavirus 2 (SARS-CoV-2): a diagnostic challenge in the coronavirus disease (COVID) era
Source: Pediatr Radiol. 2022 Apr 8;52(6):1038–47. doi: 10.1007/s00247-022-05346-2 (PMC8990674; doi:10.1007/s00247-022-05346-2)
Supplement: Supplementary file 3 — (DOCX 22.0 kb) [file 247_2022_5346_MOESM3_ESM.docx]

**Online Supplementary Material 3** Imaging findings and hospital course of children included in study

| Patient number | Modality | Appendicitis | Lymph node size (short axis) | Lymph node group (1–5=1; 6–10=2; >10=3) | Ileal thickening | Cecum thickening | Ascending colon thickening | Other bowel thickening | Bowel wall thickness | Mesenteric inflammation | Free fluid | Collection | Other imaging findings | ICU admission | 2-D echo |
| --- | --- | --- | --- | --- | --- | --- | --- | --- | --- | --- | --- | --- | --- | --- | --- |
| 1 | US | Yes, 6 mm | 18-mm short, 24-mm long | 2 | Yes | Yes | – | No | 6 mm | Yes | Yes | No | Splenomegaly | No | No |
| 2 | CT | No | 18-mm short, 26-mm long | 2 | Yes | Yes | – | No | 6 mm | Yes | No | No | No | No | Normal |
| 3 | US | No | No | 0 | No | No | No | No | No | No | Yes | No | MR brain: splenium diffusion restriction;  US: echogenic kidneys | No | Normal |
| 4 | US/ CT | Yes, 6 mm (8 mm on f/u CT) | 20-mm short, 32-mm long | 3 | Yes | Yes | – | No | 5 mm | Yes | Yes | No | No | Yes, inotropic support | Normal |
| 5 | US/ CT/ MR | Yes, 6 mm | 15-mm short, 17-mm long | 2 | Yes | Yes | – | Sigmoid | 5 mm | Yes | Yes | Yes | Collection, 6 mm appendicolith, enlarged kidneys | No | Normal |
| 6 | US / CT | Yes, 9 mm | 15-mm short, 24-mm long | 3 | Yes | Yes | Yes | Esophagus, proximal ileum | 7 mm | Yes | Yes | No | No | No | Systolic dysfunction |
| 7 | CT | Yes, 7 mm | 15-mm short, 15-mm long | 2 | Yes | No | – | No | 5 mm | Yes | Yes | No | No | No | Enlarged ventricles, no aneurysm |
| 8 | US | No | No | 0 | No | No | No | No | No | No | No | No | No | Yes, inotropic support | Systolic dysfunction |
| 9 | US | No | No | 0 | No | No | No | No | No | No | Yes | No | No | Yes, inotropic support | Systolic dysfunction, dilated coronaries, no aneurysm |
| 10 | US | No | No | 0 | No | No | No | No | No | No | Yes | No | No | Yes, inotropic support | Normal |
| 11 | CT | No | 5-mm short, 5-mm long | 1 | No | Yes | No | No | No | No | No | No | Splenomegaly | No | 4-mm left coronary dilation |
| 12 | CT | Yes, 9 mm | 8.5-mm short, 15-mm long | 1 | No | No | No | No | No | Yes | Yes | No | Splenomegaly | No | Thin rim of pericardial fluid |
| 13 | US | No | 5-mm short, 5-mm long | 1 | No | No | No | No | No | Yes | Yes | No | No | No | Normal |
| 14 | US | No | No | 0 | No | No | No | No | No | No | Yes | No | No | Yes, inotropic support | Systolic dysfunction |
| 15 | US | No | No | 0 | No | No | No | No | No | No | Yes | No | Bilateral GGO/ consolidation on initial CT chest | Yes inotropic, ventilator support | Systolic dysfunction |
| 16 | US | No | No | 0 | No | No | No | Yes, left flank small bowel | 3 mm | No | Yes | No | RCA and left diagonal aneurysm on CT as well | Yes | RCA and left D1 aneurysm |
| 17 | US | No | No | 0 | No | No | No | No | No | No | Yes | No | No | Yes | Normal |
| 18 | US | No | No | 0 | No | No | No | No | No | No | No | No | Focal renal heterogeneous echogenicity, normal f/u US | No | Mild coronary dilation, 4 mm |
| 19 | US | No | 7-mm short, 8-mm long | 1 | No | No | No | No | No | Yes | No | No | Splenomegaly | No | Normal |
| 20 | CT | No | 7-mm short, 10-mm long | 1 | Yes | Yes | No | No | 7 mm | Yes | Yes | No | Pericholecystic fluid | Yes, inotropic support | Systolic dysfunction |
| 21 | US | No | No | 0 | No | No | No | No | No | No | Yes | No | No | Yes, inotropic support | Normal |
| 22 | US | No | 10-mm short, 16-mm long | 1 | No | No | No | No | No | Yes | Yes | No | Enlarged kidneys on f/u scan | Yes, inotropic support | Normal |
| 23 | US/ CT | Yes, 7 mm | No | 0 | Yes | Yes |  | Yes, small bowel | 4.5 mm | Yes | Yes | No | Kidney enlargement >1 cm change on CT 4 days after US | No | No |

*D1* 1^st^ diagonal branch, *f/u* follow-up, *GGO* ground-glass opacity, *ICU* intensive care unit, *RCA* right coronary artery
