# Supplementary material for: Appendiceal involvement in pediatric inflammatory multisystem syndrome temporally associated with severe acute respiratory syndrome coronavirus 2 (SARS-CoV-2): a diagnostic challenge in the coronavirus disease (COVID) era
Source: Pediatr Radiol. 2022 Apr 8;52(6):1038–47. doi: 10.1007/s00247-022-05346-2 (PMC8990674; doi:10.1007/s00247-022-05346-2)
Supplement: Supplementary file 4 — (DOCX 18.5 kb) [file 247_2022_5346_MOESM4_ESM.docx]

**Online Supplementary Material 4** Laboratory results of all the children included in study

| Pt. No. | CRP (0.5–5 mg/L) | Lymphopenia (1.5–5) | Neutrophilia | Fibrinogen (1.7–4.2 g/L) | Ferritin (13–150 ug/L) | Pro-BNP (22–157) | D-dimer (0–500) | Troponin T (0–14) | Hypoalbum-inemia (30–45) | RT-PCR (swab) | COVID-antibody |
| --- | --- | --- | --- | --- | --- | --- | --- | --- | --- | --- | --- |
| 1 | 87 | 2.98 | 9.7 | 5.9 | 271 | 249 | 1,690 | 4 | 34 | Negative | Negative |
| 2 | 185 | 1 | 20.6 | 6.6 | 677 | 1,911 | 3,860 | 14 | 21 | Negative | Positive |
| 3 | 148 | 0.5 | 6.9 | 5.1 | 1,035 | 1,729 | 7,014 | 53 | 21 | Negative | Not done, mum COVID positive 6 weeks ago |
| 4 | 344 | 0.41 | 13.8 | 6.6 | 2,316 | 22,783 | 6,992 | 45 | 14 | Negative | Positive |
| 5 | 289 | 1.27 | 14.7 | 6.4 | 186 | 610 | 5,776 | 3 | 27 | Negative | Positive |
| 6 | 343 | 0.25 | 6.8 | 4.9 | 1,795 | 17,485 | 7,746 | 34 | 19 | Negative | Positive |
| 7 | 347 | 1.14 | 10.06 | 7 | 803 | 4,500 | 4,084 | 89 | 21 | Negative | Positive |
| 8 | 238 | 0.78 | 11.4 | 4.7 | 1,617 | 7,315 | 2,739 | 163 | 18 | Negative | Not done |
| 9 | 109 | 1.09 | 29.6 | 5.9 | 679 | 3,901 | 3,726 | 39 | 21 | Negative | Positive |
| 10 | 240 | 0.82 | 8.9 | 6 | 395 | 70,000 | 3,542 | 107 | 17 | Negative | Positive |
| 11 | 330 | 0.72 | 6.6 | 5.9 | 185 | 5,413 | 1,335 | 188 | 27 | Negative | Positive |
| 12 | 105 | 2.53 | 6.43 | 0.9 | 12,378 | 1,465 | 80,000 | 5 | 25 | Negative | Negative |
| 13 | 66 | 0.32 | 10.75 | –- | 162 | 97 | 4,203 | 3 | 27 | Negative | Negative |
| 14 | 202 | 1.6 | 9.35 | 5.1 | 1,074 | 22,368 | 8,346 | 28 | 22 | Positive | Positive |
| 15 | 206 | 1.45 | 34.9 | 4.5 | 1,553 | 7,343 | 9,769 | 22 | 14 | Negative | Positive |
| 16 | 219 | 0.28 | 7.3 | – | 271 | 63 | – | 3 | 27 | Negative | Negative |
| 17 | 239 | 1.39 | 2.57 | – | 514 | 17,862 | 7,545 | 9 | 23 | Negative | Negative |
| 18 | 203 | 0.83 | 12.1 | 6.9 | 421 | 281 | 1,812 | 4 | 23 | Negative | Negative |
| 19 | 107 | 1.34 | 19.8 | 5.4 | 356 | 4,575 | 1,405 | 26 | 18 | Negative | Positive |
| 20 | 158 | 1.26 | 13.7 | 1.2 | 1,709 | 70,000 | 17,328 | 104 | 17 | Negative | Negative |
| 21 | 215 | 0.43 | 8.8 | 6.9 | 1,556 | 2,200 | 6,112 | 17 | 27 | Negative | Positive |
| 22 | 142 | 0.53 | 3.58 | 5.7 | 354 | 11,675 | 2,733 | 9 | 22 | Negative | Not done |
| 23 | 274 | 1.1 | 12.1 | 9.4 | 197 | 30 | 12,982 | 4 | 26 | Negative | Positive |

*PCR* polymerase chain reaction *Pt. No*. Patient Number
