# Supplementary material for: Appendiceal involvement in pediatric inflammatory multisystem syndrome temporally associated with severe acute respiratory syndrome coronavirus 2 (SARS-CoV-2): a diagnostic challenge in the coronavirus disease (COVID) era
Source: Pediatr Radiol. 2022 Apr 8;52(6):1038–47. doi: 10.1007/s00247-022-05346-2 (PMC8990674; doi:10.1007/s00247-022-05346-2)
Supplement: Supplementary file 5 — (DOCX 14.1 kb) [file 247_2022_5346_MOESM5_ESM.docx]

**Online Supplementary Material 5** Summary of laboratory results of the 23 children with pediatric inflammatory multisystem syndrome (PIMS-TS) from our study

| Laboratory values  (normal range) | Median (IQR) | % abnormal |
| --- | --- | --- |
| C-reactive protein  (0.5–5 mg/L) | 205 (145–239) | 23/23, 100% |
| Lymphocytes, x10^9/L  (1.5–5 x10^9/L) | 1.0 (0.5–1.3) | 22/23, 96% |
| Neutrophils, x10^9/L  (1.5–7 x10^9/L) | 10.1 (7.1–13.8) | 19/23, 83% |
| Ferritin  (13–150 ug/L) | 677 (313–1,555) | 22/23, 96% |
| Fibrinogen^a^  (1.7–4.2 g/L) | 5.9 (5.1–6.6) | 20/20, 100% |
| D-dimer^b^  (0–500 ng/mL) | 4,990 (2,740–7,696) | 22/22, 100% |
| Albumin  (30–45 g/L) | 22 (18.3–26.5) | 22/23, 96% |
| ProBNP pg/mL  (22–157) | 4,500 (1,037–14,580) | 20/23, 87% |
| Troponin T  (0–14 ng/L) | 22 (4.5–49) | 14/23, 61% |

*IQR* interquartile range

^a^ Fibrinogen was not tested acutely in 3 patients

^b^ D-dimer was not tested acutely in 1 patient
