# Supplementary material for: Appendiceal involvement in pediatric inflammatory multisystem syndrome temporally associated with severe acute respiratory syndrome coronavirus 2 (SARS-CoV-2): a diagnostic challenge in the coronavirus disease (COVID) era
Source: Pediatr Radiol. 2022 Apr 8;52(6):1038–47. doi: 10.1007/s00247-022-05346-2 (PMC8990674; doi:10.1007/s00247-022-05346-2)
Supplement: Supplementary file 6 — (DOCX 17.3 kb) [file 247_2022_5346_MOESM6_ESM.docx]

**Online Supplementary Material 6** Results from 10 studies included in detailed data analysis as per review study selection criteria

| Study (author) | RT-PCR or serological SARS-CoV-2 | Imaging or surgical findings | Imaging findings |
| --- | --- | --- | --- |
| Blumfield et al.  *n*=16 | 3 – RT-PCR  10 – serology  1 – RT-PCR and serology  2 – all negative | 8 US abdomen  2 CT abdomen | US  75% Mild ascites  75% hepatomegaly  63% echogenic kidneys  38% GB wall thickening  13% Splenomegaly  CT showed additional:  Small bowel and colon wall thickening in 1 patient, and enlarged mesenteric lymph node in 2 patients (size not mentioned) |
| Dufort et al.  *n*=99  (abdominal symptoms 44) | 20% RT-PCR  80% serology | Of 44 patients, 34 (77%) had CT abdomen, US abdomen, or both | 36% ascites or pelvic fluid  18% mesenteric lymphadenopathy  16% bowel wall thickening  11% gallbladder inflammation  9% hepatomegaly, splenomegaly or hepatosplenomegaly  5% inflammation or enlargement of appendix |
| Fenlon et al.  *n*=47 | 55% serology  28% RT-PCR and serology  9% RT-PCR  (0–21 years of age) | 13 abdominal US/  2 MRI | 54% (7/13) ascites  23% (3/13) RLQ bowel thickening  17% (2/13) rectosigmoid thickening  23% (3/13) gallbladder wall thickening  17% (2/13) abdominal lymph nodes  8% (1/13) appendix thickened 7 mm |
| Hameed et al.  *n*=35 | 90% anti-bodies positive | 19 US abdomen  5 CT abdomen | US abdomen  53% ascites  47% RIF mesenteric inflammation  47% enlarged mesenteric lymph nodes (>3, 8–15 mm)  16% terminal ileum thickening  16% cecal thickening  16% mild gallbladder wall thickening, gallbladder sludge, pericholecystic edema  9% hepatomegaly and splenomegaly  9% splenic lesions  4% echogenic kidneys |
|  |  |  | CT abdomen  80% ascites  60% mesenteric fat inflammation and enlarged LN (2 also seen on USG)  40% distal ileal and caecal thickening  40% pericholecystic edema  20% splenic infarct |
| Lee et al.  *n*=28 | 61% RT-PCR  63% serology | 6 US | 33% mesenteric adenitis  17% mild ascites  17% increased liver echogenicity  17% increased kidney echogenicity |
| Mamishi et al.  *n*=45 | 22% RT-PCR  78% serology | No mention of number of US/CT scan | 11% ascites  4% ileitis |
| Miller et al.  *n*=44 | 15 RT-PCR  31 serology | 12 US  1 CT  2 MRI | 40% ascites/free fluid  20% ileitis  20% gallbladder wall thickening  20% gallbladder sludge  13% mesenteric adenitis RIF  13% hepatomegaly/altered liver echogenicity  6% rectosigmoid thickening  6% mesenteric fat inflammation  6% pericholecystic fluid  6% prominent appendix |
| Riphagen et al.  *n*=8 | All RT-PCR negative | Imaging (modality not mentioned) | 71% ascites  14% gallbladder edema  14% ileitis |
| Toubiana et al.  *n*=21 |  | Imaging not mentioned | 4 ascites |
| Tullie et al.  *n*=8 | 5 RT-PCR  Ab not performed | 8 US  4 CT | 87% enlarged mesenteric lymph node  62% terminal ileitis  37% mesenteric inflammation  12% free fluid  12% cecum thickening |

*Ab antibody test*, *RIF* right ileac fossa, *RLQ* right lower quadrant, *RT-PCR* reverse transcriptase-polymerase chain reaction, *SARS-CoV-2* severe acute respiratory syndrome coronavirus 2
