# Supplementary material for: Appendiceal involvement in pediatric inflammatory multisystem syndrome temporally associated with severe acute respiratory syndrome coronavirus 2 (SARS-CoV-2): a diagnostic challenge in the coronavirus disease (COVID) era
Source: Pediatr Radiol. 2022 Apr 8;52(6):1038–47. doi: 10.1007/s00247-022-05346-2 (PMC8990674; doi:10.1007/s00247-022-05346-2)
Supplement: Supplementary file 7 — (DOCX 16.4 kb) [file 247_2022_5346_MOESM7_ESM.docx]

**Online Supplementary Material 7** Additional 10 studies from literature search that did not fulfil review inclusion criteria but mentioned results of abdominal imaging or intraoperative findings in pediatric inflammatory multisystem syndrome (PIMS-TS)/multisystem inflammatory syndrome in children

| Study (author) | RT-PCR or serological SARS-CoV-2 | Imaging or surgical findings | Imaging findings |
| --- | --- | --- | --- |
| Cheung et al.  *n*=17 | 8 RT-PCR  9 serology | Imaging modality not mentioned | Acute ileocolitis (*n*=1) on imaging |
| Dallan et al.  *n*=2 | 2 +ve serology | 2 CT abdomen | 1 ileocolitis, terminal ileitis and appendicitis  1 enlarged mesenteric lymph nodes (size not mentioned) |
| Dhanalakshmi et al.  *n*=19 | 4 RT-PCR  5 serology  4 not tested | 5 US abdomen  1 CT abdomen | Suspicious appendicitis on US/CT (*n*=1)  No other imaging details |
| D’souza et al.  *n*=17 | 15/15 negative RT-PCR nasal swab | US | Acute appendicitis (*n*=1); managed conservatively because other tests favored PIMS-TS |
| Harwood et al.  *n*=2 | Pt 1: –ve RT-PCR  Pt 2: +ve RT-PCR (but treated as appendicitis) | 1 US  1 CT | US: mesenteric lymph nodes  CT: perforated appendicitis with appendicolith (operated and settled with antibiotics) |
| Feldstein et al.  *n*=131 | 73 RT-PCR  58 serology | Modality not mentioned | 10% hepatitis/hepatomegaly  2% gallbladder hydrops  1% appendicitis |
| Jackson et al.  *n*=1 | RT-PCR negative  Serology positive | Not mentioned | Acute appendicitis (9 mm) and ileal thickening  Operated: appendectomy and inflamed ileal R/A and necrotic mesenteric lymph nodes  Post-op: developed collection and thickening of cecum/ ascending colon. Treated as MISC and all resolved on follow-up  Histopathology inconsistent with acute appendicitis. Acute vasculitis features in excised mesentery |
| Lishman et al.  *n*=4 | 4 cases of acute appendicitis  4 RT-PCR positive | No imaging.  3 patients underwent surgery | All 3 underwent surgery for appendicitis and post-op diagnosed as MIS-C  2 perforated appendicitis with pus  1 peritonitis  1 mesenteric lymphadenitis  No appendicoliths.  No mention of ileal/caecal involvement |
| Patel et al.  *n*=1 | RT-PCR negative | CT scan and US | CT (day 1) – enlarged mesenteric lymph nodes, normal appendix  US (day 7) – enlarged lymph nodes in RIF with free fluid |
| Waltuch et al.  *n*=4 | 4 serology  All RT-PCR negative | US and CT scan | 1 US – gallbladder wall edema and mild ascites  1 CT – mild appendix thickening and wall enhancement with mild diffuse colon wall thickening |

*RIF* right ileac fossa, *RT-PCR* reverse transcriptase-polymerase chain reaction, *SARS-CoV-2* severe acute respiratory syndrome coronavirus 2, *+ve* positive, *–ve* negative
