# Supplementary material for: Appendiceal involvement in pediatric inflammatory multisystem syndrome temporally associated with severe acute respiratory syndrome coronavirus 2 (SARS-CoV-2): a diagnostic challenge in the coronavirus disease (COVID) era
Source: Pediatr Radiol. 2022 Apr 8;52(6):1038–47. doi: 10.1007/s00247-022-05346-2 (PMC8990674; doi:10.1007/s00247-022-05346-2)
Supplement: Supplementary file 8 — (DOCX 16.6 kb) [file 247_2022_5346_MOESM8_ESM.docx]

**Online Supplementary Material 8** Data on COVID-19 testing and percentage of patients undergoing imaging from analysis of relevant included studies (*n*=10) in literature review. *RT-PCR* reverse transcriptase-polymerase chain reaction
